# Brain Hypothermia Therapy and Targeted Temperature Management for Acute Encephalopathy in Children: Status and Prospects

**DOI:** 10.3390/jcm12062095

**Published:** 2023-03-07

**Authors:** George Imataka, Yuji Fujita, Jin Kikuchi, Koji Wake, Kazuyuki Ono, Shigemi Yoshihara

**Affiliations:** 1 Department of Pediatrics, Dokkyo Medical University, 880 Kitakobayashi, Mibu, Shimotsuga, Tochigi 321-0293, Japan; 2 Department of Emergency and Critical Care Medicine, Dokkyo Medical University, 880 Kitakobayashi, Mibu, Shimotsuga, Tochigi 321-0293, Japan

**Keywords:** brain hypothermia therapy, target temperature management, neuronal protection, hibernation

## Abstract

In adult intensive care, brain hypothermia therapy (BHT) was reported to be effective in neuroprotection after resuscitation and cardiac arrest. By contrast, in neonatal intensive care, the pathophysiology of brain damage caused by hypoxic–ischemic encephalopathy (HIE) is attributed to circulatory disturbances resulting from ischemia/reperfusion, for which neonatal brain cryotherapy is used. The *International Liaison Committee on Resuscitation*, 2010, recommends cerebral cryotherapy for HIE associated with severe neonatal pseudoparenchyma death. The usefulness of BHT for neuroprotection in infants and children, especially in pediatric acute encephalopathy, is expected. Theoretically, BHT could be useful in basic medical science and animal experiments. However, there are limitations in clinical planning for treating pediatric acute encephalopathy. No international collaborative study has been conducted, and no clinical evidence exists for neuroprotection using BHT. In this review, we will discuss the pathogenesis of neuronal damage in hypoxic and hypoperfused brains; the history of BHT, its effects, and mechanisms of action; the success of BHT; cooling and monitoring methods of BHT; adverse reactions to BHT; literature on BHT. We will review the latest literature on targeted temperature management, which is used for maintaining and controlling body temperature in adults in intensive care. Finally, we will discuss the development of BHT and targeted temperature management as treatments for pediatric acute encephalopathy.

## 1. Introduction

Acute encephalopathy in childhood and adolescence refers to a pathobiological condition of the brain that progresses rapidly. It is characterized by central nervous system (CNS) dysfunction caused by diffuse or widespread noninflammatory cerebral edema [1]. According to the recommendations of the Japanese Society of Child Neurology for acute encephalopathy in children and infants, the impairment of consciousness must last for at least 24 h and have a Glasgow coma score of 11 or less [2].

Several encephalopathy disorders are multifactorial, whereas others, such as previous viral infections or hepatic or uremic encephalopathies, have a well-known etiology. The influenza virus is the most common pathogen in Japan, followed by human herpesvirus 6, rotavirus, and respiratory syncytial virus. In recent years, the number of cases of acute encephalopathy associated with other viruses, such as human metapneumovirus, rhinovirus, and cytomegalovirus, has increased [3,4]. In acute encephalopathy syndrome, acute encephalopathy with biphasic seizures and late reduced diffusion (AESD) was the most common type (29%), Middle East respiratory syndrome (MERS) was the second most common type (16%), and acute necrotizing encephalopathy (ANE) was the least common type (4%). Other syndromes that occurred in 2% of cases included hemorrhagic shock encephalopathy syndrome (HSES) [3]. Although having a lower annual incidence, the number of cases of acute encephalopathy in Japan is significantly higher than in Western countries [5]. There have also been several reports of children infected with COVID-19 developing encephalitis, ANE, acute disseminated encephalomyelitis (ADEM), cytotoxic lesion of the callosal splenium, posterior reversible encephalopathy syndrome (PRES), and other neurological illnesses. 

The primary pathomechanism of acute encephalopathy is classified into four categories: metabolic error, cytokine storm, excitotoxicity, and unknown mechanisms. Noninflammatory brain edema is a pathologic feature of acute encephalopathy. Noninflammatory brain edema raises intracranial pressure, resulting in decreased cerebral perfusion pressure, and eventually, herniation syndromes and brainstem dysfunction caused by CNS-mediated respiratory and circulatory failures [6,7,8]. Whatever the cause of encephalopathy, all cases of acute encephalopathy have at least one symptom—an altered mental state. The altered mental state can be subtle and develop gradually, such as apraxia, including the inability to sketch simple drawings, or it can be obvious and develop rapidly, leading to coma or death within minutes. Inattention, poor judgment, and poor motor coordination are symptoms of disturbed mental status. Seizures are common in many people, and they are frequently febrile and last for an extended period (febrile status epilepticus). Febrile status convulsions, status febrile seizures, and status febrile epilepticus associated with fever are triggers for the increasingly reported AESD [9], which is one of the common types of acute encephalopathy. However, there is little evidence for identifying and treating acute encephalopathy, despite the relatively high rate of morbidity and mortality linked with the condition [6]. Moreover, neurological complications are also common in coronavirus disease 2019 (COVID-19) patients. COVID-19-associated multisystem inflammatory syndrome in children (MIS-C) can cause cerebrovascular events as well as abnormal eye movements. There have also been several reports of children infected with COVID-19 developing encephalitis, ANE, ADEM, and cytotoxic lesion of the corpus callosum [10,11,12,13,14]. 

The clinical course of metabolic errors and inherited metabolic disorders can include gradually progressive or static features, followed by the emergence of an acute encephalopathic crisis, which can include lethargy, behavioral changes, or gait disturbances caused by infections or fasting [15]. Specific therapies such as corticosteroids, immunoglobulin, free-radical scavengers, osmotic agents, immunosuppressants, plasmapheresis, and therapeutic hypothermia have been studied extensively [16,17,18,19,20,21]. In this review, we offer an updated summary of available data and a perspective on targeted hypothermia-based management of pediatric acute encephalopathy.

We conducted a literature search from 1 September 2000 to 1 September 2022, using the MEDLINE/PubMed and National Institutes of Health Clinical Trials Registry (http://www.clinicaltrials.gov, accessed on 1 September 2022) electronic medical databases for the identification of publications on the targeted hypothermia-based treatment of acute encephalopathy. Articles were included if they were published in the English language. We excluded conference posters. Keywords included brain hypothermia, targeted temperature management, therapeutic hypothermia, induced hypothermia, acute encephalopathy, pediatric, children, preschool, newborn, infant, acute febrile encephalopathy, and status epilepticus. Abstracts and full-text articles of randomized clinical trials, reviews, and other study designs were considered from studies describing relevant data on pediatric acute encephalopathy. An additional search was carried out via Google Scholar, and relevant articles on prospective and retrospective designs and real-world data on pediatric acute encephalopathy were considered.

## 2. Therapeutic Hypothermia

Hyperthermia is thought to be an independent poor prognostic factor in acute encephalopathy. Accordingly, numerous pharmacologic and nonpharmacologic strategies, such as targeted temperature management (TTM), have been reported when general anesthesia medication fails [22,23,24,25,26]. Neonatal brain hypothermia therapy (BHT) is cooling of the head only. As brain temperature in acute encephalopathy has been reported to be 1 °C–2 °C higher than core temperature, children were subjected to BHT for 48 h using the whole-body cooling system to set the body temperature at 33.5 °C–35 °C [23]. Since the body surface area of the head becomes smaller as the child grows, head cooling and whole-body cooling are used together to lower the body temperature more efficiently. Treatment for mild BHT that keeps the body temperature at 34.5 °C–36 °C has gained attention in recent years [27]. Moreover, hospitals have developed a BHT strategy for treating acute encephalitis or encephalopathy and status epilepticus in children. Regarding the long-term effects of AESD, hypothermia therapy may be helpful in preventing the emergence of postencephalopathic epilepsy, thereby improving patients’ quality of life [26]. 

BHT is effective in neonates due to their large head surface area while adolescents may need whole-body cooling as an effective measure. Eventually, the field of emergency medicine advocated TTM, which is controlled at a temperature not as low as BHT. TTM is defined as temperature control (34.5 °C–37 °C) with intubation and continuous use of anticonvulsants and muscle relaxants used within 24 h of onset [21]. According to numerous case reports and retrospective investigations, the neurological outcomes of acute encephalitis or encephalopathy can be greatly improved by focused TTM [2,21]. TTM is often used in neurocritical care to minimize secondary neurologic injury and improve outcomes. TTM is best supported by evidence from neonatal hypoxic–ischemic encephalopathy (HIE) and out-of-hospital cardiac arrest, although it has also been studied in patients with ischemic stroke, traumatic brain injury, and intracranial hemorrhage [28,29]. BHT and TTM are often used synonymously in pediatric practice. However, there are key differences in their approaches. Table 1 presents the summary of BHT and TTM. Mild BHT, followed by TTM, may be an effective way to improve neurological outcomes in children with HSES [27]. Although therapeutic hypothermia has been efficient, finding a solution to side effects such as bradycardia, low blood pressure, and abnormal coagulation has proven difficult [30]. 

## 3. Brief History of Therapeutic Hypothermia

Since ancient times, humans have known the use of alternative medicines that soothe inflammation by cooling injury and fever. Hypothermia was utilized medically by the Egyptians 5000 years ago. It was first mentioned in the Edwin Smith Papyrus, an ancient medical treatise on medicines and surgery written around 3500 B.C.E. Hippocrates, who advocated burying injured troops in the snow, introduced the concept that cooling delayed biological processes, and ultimately, death. Cooling the body as a therapeutic intervention became more widespread in the 17th century when physicians such as John Floyer (1649–1734) began experimenting more widely with the use of hot and cold water in medicine. The use of cryoanalgesia for amputations around the 1800s led surgeons to discover that the analgesic effects of cryoanalgesia were accompanied by a reduction in bleeding. Local head cooling for traumatic brain injury was employed in the late 1800s, and total body cooling was documented for the first time during World War II to treat head injuries. Clinical investigations using very deep hypothermia were started in the 1950s and 1960s but discontinued due to negative outcomes soon after. In three cardiac arrest cases treated with mild hypothermia in the 1990s following successful resuscitation, all three patients recovered fully and without any lasting neurological damage [31,32]. After two prospective randomized controlled trials were published in 2002 that showed significant improvements in neurological outcomes and short- and long-term survival, therapeutic hypothermia gained major interest [28].

## 4. Pathogenesis of Neuronal Damage in Hypoxic and Hypoperfused Brain Tissue

In addition to a normal environment, the brain requires optimal pH, temperature, blood flow, and osmolarity to execute its functions. However, any small disturbances in this complex system highly impact the functioning of the brain. One of the presumptive etiologies in encephalopathy is impaired glucose or oxygen delivery to the brain. Encephalopathy, and eventually, coma, can occur due to a significant decrease in substances delivery to the brain. Hypoxia, especially hypoxia–ischemia, can permanently damage the sensitive regions of the brain, including the thalamus, hippocampus, and cerebellum. The magnitude and irreversibility of impairment depend on the degree and duration of hypoxia or reduced cerebral blood flow. HIE, typically triggered by severe hypotension or cardiac arrest, is caused by the neuronal ischemic injury cascade, which involves excitatory amino acid release, intracellular calcium influx, lipid peroxidation, and cell death [28,33,34].

The hallmark of acute hypoxic brain injury (HBI) is encephalopathy, a neurologic syndrome characterized by abnormalities in consciousness, tone, and autonomic control. HBI is a clinical condition caused by a reduction in brain blood supply and oxygenation. The brain derives 95% of its energy from both glucose and oxygen through oxidative metabolism. HBI is a significant cause of mortality and morbidity in children. Hypoxic–ischemic brain injury is caused by intercellular and intracellular events that happen before, during, and after the imbalance of these substrates and their utilization in the brain. Neurons start to rapidly deteriorate immediately after HBI, possibly as a result of a cell death process marked by necrosis, loss of acute plasma membrane integrity, and adenosine triphosphate (ATP) depletion. In the second stage, neurons begin to degenerate within hours to days, mostly through a cascade of an active, strictly controlled intracellular process called apoptosis. ATP synthesis within cells is reduced when blood flow is stopped. As a result, energy-dependent ion channels become dysfunctional, which adds to intracellular sodium buildup and cytotoxic edema. Continuous ischemia causes the production of the excitatory neurotransmitter glutamate, which stimulates calcium influx by activating N-methyl-D-aspartate (NMDA) receptors [28,33,34,35]. Studies on the human fetus have reported that the NMDA receptor is expressed by at least 22 weeks of gestation [36]. By triggering lytic enzymes, accelerating the generation of free radicals, and compromising mitochondrial function, calcium influx aggravates neuronal damage. Excitotoxicity ultimately results in cell death. The neuronal ischemic injury cascade, which includes the release of excitatory amino acids, intracellular calcium influx, lipid peroxidation, and cell breakdown, is the most common cause of HIE. Multiple studies have shown that immediately treating severely neurologically impaired survivors of cardiac arrest with mild hypothermia (33 °C–36 °C for 12–24 h) has a beneficial effect.

## 5. Mechanisms of Hypothermia

More than 50 years ago, evidence of the first uncontrolled cooling for resuscitation studies was published. Briefly, in uncontrolled studies conducted in the 1950s and 1960s, infants who were not breathing spontaneously at 5 min after birth were immersed in cold water until respiration started and then allowed to spontaneously rewarm over many hours, due to the apparent protective effects of hypothermia. In nearly 200 asphyxiated newborns, the outcomes were reported to be superior to historical controls [37,38].

The latent or early recovery phase of HBI represents the most important window of opportunity for intervention. Two pioneering trials in Europe and Australia translated animal model research in therapeutic hypothermia to humans. The exact mechanisms of hypothermic neuroprotection remain unknown. However, hypothermia is hypothesized to reduce ischemic tissue damage by affecting metabolism—with each degree Celsius below normal, oxygen consumption falls by 7%, almost linearly. Due to numerous neuroprotective processes, TTM has been demonstrated to be useful for treating various types of brain traumas. These mechanisms include lowering metabolic rate to balance oxygen supply and demand; lowering the levels of excitatory neurotransmitters, such as glutamate; preventing ATP depletion; and lowering intracellular calcium overload to prevent neuronal apoptosis. Additionally, hypothermia suppresses free-radical toxicity, has favorable effects on intracellular mediator systems, and reduces intracellular acidosis [39,40].

Increasing evidence suggests that hypothermia plays a role in apoptosis suppression, especially in the developing brain. Hypothermia initiated after severe hypoxia–ischemia, for example, reduced apoptotic cell death but not necrotic cell death, and hypothermic neuroprotection in preclinical studies was closely associated with suppression of activated caspase-3. When considered collectively, these experimental data reveal that if initiated promptly, within approximately 6 h of hypoxic–ischemic injury, a long duration of mild to moderate BHT could improve long-term outcomes.

**Figure 1:**
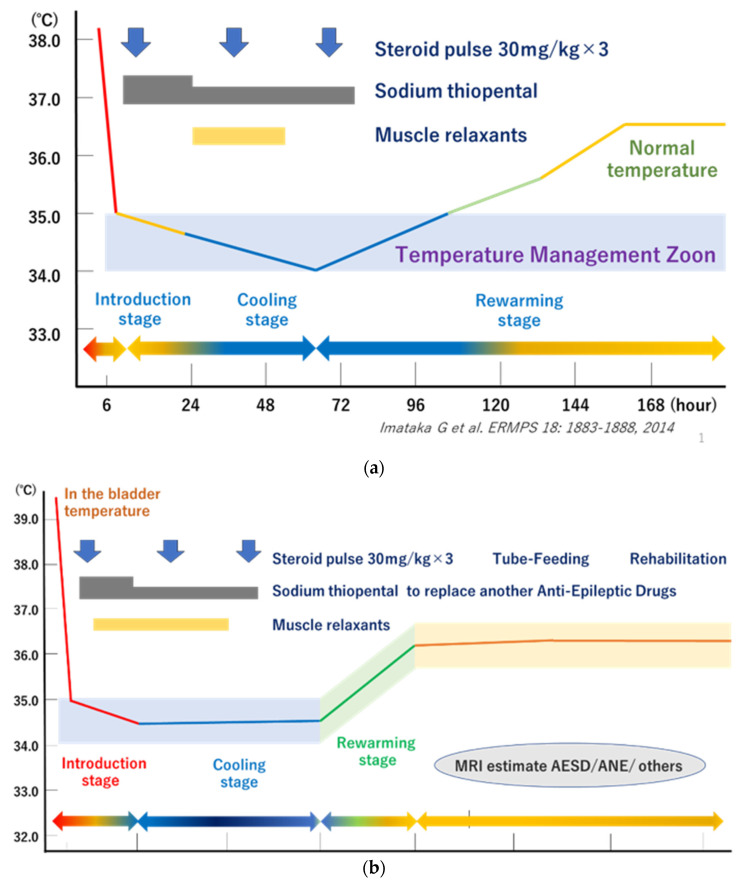
(**a**) Process for mild-brain hypothermia therapy with steroid pulse therapy [24]. (**b**) Procedure from mild-brain hypothermia therapy to targeted temperature management. AESD: Acute encephalopathy with biphasic seizures and late reduced diffusion; ANE: Acute necrotizing encephalopathy.

Clinical hypothermia is typically categorized on a scale of 0–4: normothermia (35.0 °C–37.0 °C); mild hypothermia (35.0 °C–32.0 °C); moderate hypothermia (32 °C–28 °C); severe hypothermia (below 28.0 °C). The urinary bladder is heated to 34.5 °C to maintain body temperature. After 48 h of continued hypothermia, the body temperature begins to rise at a rate of 0.5 °C every 12 h. The concurrent administration of mannitol and anesthetics is applied while administering steroid pulse therapy. A flowchart of the BHT to TTM method is shown in Figure 1. BHT is performed in three stages. The first stage is the introduction stage, followed by the cooling stage and the rewarming stage. The aim is to maintain the temperature of the urinary bladder at 34.5 °C for 48 h. Respiration, electrocardiogram, central venous pressure, and oxygen saturation are monitored concurrently during BHT. If necessary, a portable electroencephalogram is available, along with a scalp skin oxygen saturation device (INVOS), a bispectral index (BIS) monitor, and an intracranial pressure monitor.

## 6. Evidence of Therapeutic Hypothermia in Acute Encephalopathy

Clinical studies highlight the efficacy of BHT for pediatric acute encephalopathy. However, high-quality studies, including a large number of cases, demonstrate that the efficacy and safety of therapeutic techniques used in BHT for pediatric acute encephalopathy are limited. These studies have mainly focused on BHT and reported on acute encephalopathy in adults after cardiopulmonary resuscitation associated with out-of-hospital cardiac arrest and caused by ventricular arrhythmia and HIE in infants.

Especially in newborns and young children, hypoxic–ischemic encephalopathy caused by asphyxia is linked to high mortality and long-term neurodevelopmental impairment. Data from 11 RCTs (N = 1505 term and late preterm infants) were compiled in a recent Cochrane review. According to the review, TTM helped term babies who had HIE. In term neonates born with HIE, the risk of death or severe disability in the form of neurodevelopmental disability was reduced. The death rate of newborns with this condition can be decreased using both selective head cooling and whole-body cooling techniques. Mild TH (33.5 °C–34.5 °C for 72 h) is used to treat term babies (35-week gestation) with hypoxic–ischemic insult in the perinatal phase [41]. According to the results of a randomized trial (N = 168 infants), hypothermia started between 6 and 24 h after birth had a 76% chance of reducing death or disability among term infants with HIE and a 64% chance of reducing death or disability by at least 2% by the time they were 18–22 months old, compared with when no cooling techniques were used [42]. Evidence indicates lowering temperature is the most economical intervention with better outcomes for neonates with severe perinatal HIE. Compared with no cooling, more neonates survive morbidity-free despite the higher cost of hypothermia intervention [43]. In several Japanese tertiary centers, therapeutic hypothermia has been used for managing brain edema in pediatric patients with brain injuries, including postcardiac arrest syndrome and acute encephalopathy associated with febrile infection, which has been extrapolated from neonatal HIE and adult postcardiac arrest syndrome.

The clinical diagnostic classification of pediatric acute encephalopathy has advanced recently, in part because of advancements in radiological diagnosis. Many of the most severe cases of pediatric acute encephalopathies, including ANE, HSES, AESD, and convulsion-overload encephalopathy, do not respond well to conventional treatment; therefore, hypothermic treatment is expected to be effective.

Based on the evidence for BHT in adults and neonates, we started using BHT in children to treat cytokine storm-related encephalopathy, which has status epilepticus as its principal symptom. In addition, we used it in cases of near-drowning based on data from neonatal HIE. We emphasized the management of the CNS and therapy in our treatment plans for acute encephalopathy and status epilepticus.

Cerebral cryotherapy is likely to be useful in delaying the development of the second phase or reducing the sequelae in AESD, where early diagnosis is challenging because of the biphasic course and frequently delayed treatment. In a retrospective analysis of 34 patients with AESD who were admitted over a 14-year period, therapeutic hypothermia was started before the second phase in patients who had more significant consciousness disturbance between 12 and 24 h following the first seizure. Catecholamine was given to most of these patients. Multivariate analysis revealed poor outcomes that were determined by the presence of lesions in the basal ganglia or thalamus in the MRI as well as by the Tada score. Third, there was a high correlation between worse outcomes and 1st time cooling in the Early-Hypo group, despite the small population and other confounding factors, but no correlation was seen between the outcomes and 1st time cooling or 2nd time cooling in the overall AESD population with TTM [44].

**Figure 2:**
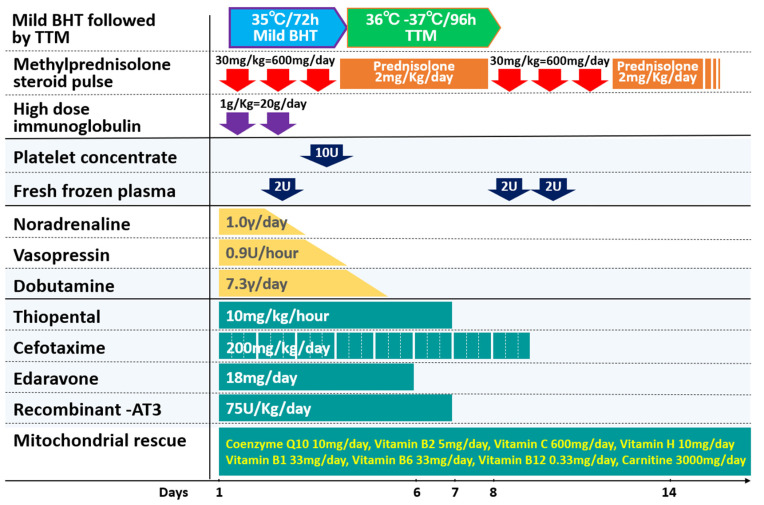
Brain hypothermia therapy is managed in the ICU followed by targeted temperature management in the ICU or general ward [27]. BHT: Brain hypothermia; ICU: Intensive care unit; TTM: Targeted temperature management. Case description: A 4-year-old patient was immediately admitted to the intensive care unit. Within 4 h of symptom onset, she received mild-brain hypothermia therapy with a target body temperature of 35 °C for 48–72 h with ICU management. Methylprednisolone pulse, high dose immunoglobulin, and large doses of circulatory drugs were administered. After 72 h of brain hypothermia therapy, targeted temperature management with a target body temperature between 36 °C and 37 °C was continued for 96 h while weaned from ventilation. Magnetic resonance imaging results revealed no signs of acute encephalopathy. She was discharged without neurological sequelae 28 d after symptom onset.

Recently, two case reports drew attention to the use of cerebral cryotherapy followed by TTM for most severe pediatric acute encephalopathy with a good prognosis. The first case of HSES was reported. The second case involved a case of multiple encephalopathy syndrome, which included multiple pediatric acute encephalopathies, such as ANE, AESD, MERS, and acute small encephalopathy. The cryotherapy protocol used in these two cases is shown in Figure 2. Both cases were treated with steroid pulse therapy, and both began with 76-h BHT, steroid pulse therapy, and gammaglobulin therapy [20,45]. TTM is then extended. This new protocol for severe pediatric acute encephalopathy, which consists of cerebral cryotherapy followed by TTM, has the potential to be a new treatment modality, and we hope to see more cases in the future.

A retrospective study of children with acute encephalopathy and serum aspartate aminotransferase levels below 90 IU/L within 6 h of onset (N = 57 children) evaluated the effectiveness of TTM. Grade 1 in *Pediatric Cerebral Performance Category Scale*, representing good clinical outcomes, was observed in 67% of the children [21]. Another retrospective study compared the clinical course and outcome in 20 children treated with TTM (36 °C) and methylprednisolone pulse (MP) therapy (TTM/MP) with those treated with conventional MP therapy in a secondary emergency care hospital. All children treated with TTM/MP survived, and their body temperature dropped quickly. Some cases of acute encephalopathy are of the so-called fulminant kind, in which DIC or shock manifests quickly after the start, making it occasionally challenging to administer TTM. Patients of the fulminant variety need to be taken to tertiary emergency care facilities. Secondary emergency care facilities must carefully choose patients for TTM while considering the likelihood of evacuation to a tertiary emergency facility [46]. Table 2 summarizes the evidence for TTM or BHT in acute encephalopathy, and Table 3 summarizes the BHT protocol at our center.

Data from clinical trials in high-income countries suggest that using induced hypothermia for moderate or severe neonatal encephalopathy reduces death or disability at 18 months [51]. When not managed properly, however, TTM can be associated with negative effects and unfavorable outcomes. In pediatric patients with head injury or postcardiac arrest, hypotension has been linked to therapeutic hypothermia, and thrombocytopenia has been linked to therapeutic hypothermia in adult patients with head injuries and infants with HIE. Among children with mild-to-severe neonatal encephalopathy, TTM was independently associated with decreased odds of abnormal motor (OR: 0.15, 95% CI: 0.06–0.40, *p* < 0.001) and cognitive (OR: 0.11, 95% CI: 0.04–0.33, *p* < 0.001) outcomes even after adjusting for propensity [52]. After neonatal encephalopathy, therapeutic hypothermia is associated with lower rates of brain injury and worse 30-month outcomes. Therapeutic hypothermia has no effect on the predictive accuracy of MRI in the first week of life. Normal MRI continues to be reassuring for a normal 30-month outcome following therapeutic hypothermia [53].

## 7. Gaps and Future Directions

In low- and middle-income countries, therapeutic hypothermia had no effect on the combined outcome of death or disability 18 months after neonatal encephalopathy, but it significantly increased death alone. Even when tertiary neonatal critical care facilities are available, therapeutic hypothermia should not be administered as a treatment for neonatal encephalopathy in low- and middle-income nations [51]. Although therapeutic hypothermia for neonatal encephalopathy reduces neurologic impairment and cerebral palsy, its impact on neonatal, infantile, and childhood mortality remains unknown according to a recent meta-analysis [52]. The environment in which it is executed has an impact on the consequences. Trials of low(er) quality overstated the potential benefit of therapeutic hypothermia. Whole-body management at the PICU level is necessary for BHT. Steroid pulse therapy, globulin therapy, and intravenous therapy of various antiseizure drugs are frequently used in conjunction with BHT in some cases (25). To confirm the clinical results of this therapy, it is necessary to evaluate the clinical course of treatment by dividing the cases into two groups based on treatment received as follows: (1) receiving BHT and (2) receiving BHT with concomitant medicines. The number of studies on this topic is limited. However, informed consent is a deciding element in BHT research.

During acute encephalopathy, cytokine release is triggered by various factors, including infection, trauma, and other insults to the brain. The excessive release of cytokines leads to an inflammatory response, which can result in brain damage and even death [54]. The rationale for using TTM for acute encephalopathy is to reduce cytokine-mediated inflammation that may be exacerbated by fever and sepsis. TTM is believed to reduce cytokine-mediated inflammation through several mechanisms. One of the primary ways TTM works is by reducing the metabolic rate of cells, which slows down the release of cytokines. The reduced metabolic rate also leads to a decrease in the production of reactive oxygen species, which are known to cause oxidative stress and contribute to inflammation. TTM has also been shown to improve blood flow to the brain, which helps reduce inflammation by increasing the delivery of oxygen and nutrients to brain cells. TTM also has neuroprotective effects, which can help prevent further damage to the brain [55,56]. In addition, the antiepileptic effect of TTM is based on various changes at the molecular level. Because TTM activates multiple anticonvulsant and neuroprotective mechanisms in the pathophysiology of acute encephalitis and refractory status epilepticus, it has the potential to impact seizure control and neuroprotection in the setting of refractory status epilepticus. Most studies on TTM for refractory status epilepticus have focused on animal models, and few clinical studies exist [57,58]. In addition, TTM may contribute to seizure control. However, in most case studies, the patients received a combination of intravenous anesthetic agents and TTM [47]; thus, the seizure response reported may reflect the combination of therapies. The benefits of TTM for refractory status epilepticus may be linked to the etiology of the seizure, and further large-scale studies are needed to evaluate refractory status epilepticus from various causes to determine which groups would benefit from TTM. TTM tends to be used in mild and moderate encephalopathies to avoid the risk of BHT treatment. However, it is not certain that the most severe acute encephalopathies can be successfully treated with TTM alone. BHT is associated with complications, such as arrhythmia, coagulopathy, electrolyte abnormalities, increased infection rates, and venous thrombosis; however, it is possible to successfully use or combine BHT and TTM in different cases to achieve a more ideal treatment for pediatric acute encephalopathies. 

## 8. Conclusions

The targeted temperature for managing acute encephalopathy depends on the underlying cause of the condition and the severity of the symptoms. The use of targeted temperature management for acute encephalopathy should be individualized to each patient’s specific needs and medical history and should only be applied under the guidance of a qualified healthcare provider. We hope to establish a more ideal systemic management of pediatric acute encephalopathy by using BHT and TTM in different or combined cases.

## Figures and Tables

**Table 1:** Distinguishing between BHT and TTM.

BHT	TTM
Head cooling alone for neonates Whole-body cooling for infants and children	Whole body mild cooling
Lower body temperature by more than 2 °C The target body temperature for BHT is 32 °C to 35 °C	Lower body temperature by 1–2 °C TTM maintains and controls the target body temperature at 36 °C for an extended period of time
Generally, ventilator management is required	Ventilator management is not required
Higher rewarming process	Relatively less duration of rewarming phase
Requires ICU support-intubation and ventilator management. Intravenous fluids, maintenance of blood pressure, and systemic circulatory control are required.	Can be performed in general wards
Antiepileptic drugs such as barbiturates and steroid pulse therapy are often used in combination in severe pediatric acute encephalopathy.	For the most severe form of acute encephalopathy in children with high-cytokine type, TTM is not sufficient for management.

BHT: Brain hypothermia; ICU: Intensive care unit; TTM: Targeted temperature management.

**Table 2:** Summary of clinical studies and case reports for targeted temperature management in acute encephalopathy [20,29,47,48,49,50].

Reference	Indication	Age (Years)	N	Goal Temp. (°C)	Duration of Hypothermia, Hours	Findings
TTM						
Guilliams et al. (2013) [47]	Refractory status epilepticus	5 months–15 years	5	32–35	24–120	No recurrence (100%). One child died. Two patients required PICU care.
Nishiyama et al. (2015) [21]	Acute encephalopathy without serum aspartate aminotransferase elevation	6.9–14.2 years	57	34.5–36	72	Good neurological outcomes (100%). PICU not required
Shankaran et al. (2005) [29]	Moderate/severe HIE	Neonates of at least 36 weeks	102 to HT 106 to control	Esophageal temperature of 33.5 for 72 h	72 h	Death or moderate or severe disability: 44% with whole-body cooling vs. 62% control. (risk ratio, 0.72; 95% CI, 0.54 to 0.95; *p* = 0.01)
Brain Hypothermia Therapy						
Kawano et al. (2011) [48]	Acute child encephalopathy and encephalitis treated at PICUs.	2–5 years	43	33.5–35	48–72	PCPC outcomes: Early HT (≤12 h): 14/17 (82.4%) versus late HT (>12 h): 2/10 (20%) versus NT: 9/16 (56.3%)
Yang et al. (2020) [49]	Moderate/severe HIE with 1-min Apgar score ≤ 3 and 5-min Apgar score ≤ 5	Neonates < 6 h	62 to HT group 30 to control group	28.0–30.0 (head skin Temp.); 34.5 ± 0.5 (body surface skin); 35.5 ± 0.5 (anal temp)	72	Hypothermia treatment of 72 h is better than 48 h for improving oxidative conditions, reducing NSE values, and improving neurological behavior
Gluckman et al. (2005) [50]	Moderate/severe HIE	Neonates < 6 h	116 to HT group 118 to control group	Rectal temperature maintained at 34–35	72 h	Head cooling had no effect on infants with the most severe aEEG changes (n = 46, 1.8; 0.49–6.4, *p* = 0.51), but was beneficial to infants with less severe aEEG changes (n = 172, 0.42; 0.22–0.80, *p* = 0.009).

aEEG: Amplitude-integrated electroencephalography; HIE: Hypoxic Ischemic Encephalopathy; HT: Hypothermia; NSE: Neuron-specific enolase; PICU: Pediatric intensive care unit.

**Table 3:** Proposed protocol for BHT followed by targeted temperature management (Dokkyo Medical University Hospital ICU: 2020).

Antiseizure medication treatment for status epilepticus (A) Midazolam (0.5 mg/kg) administered through the nasal cavity or cheek mucosa. (B) Midazolam (0.15 mg/kg) intravenously (i.v.) administered (up to two doses possible). (C) Between ages 0 and 2 years: intravenous phenobarbital; between 15 and 20 mg/kg (10 min i.v.), ≥2 years: phenobarbital or fosphenytoin 22.5 mg/kg (10 min i.v.). If febrile convulsions associated with fever and Dravet syndrome cannot be ruled out, phenobarbital should be used instead of fosphenytoin. (D) Sodium thiopental between 3 and 5 mg/kg (slow i.v.). Brain hypothermia therapy This protocol applies to infants weighing ≥7.5 kg and aged ≥6 months. Introductory period 1. Status epilepticus/acute encephalopathy admission: ICU (request for admission), contact brainwave department or radiology (brain and chest CT). 2. Check vital signs and establish a peripheral line. 3. Establish central venous line: establish double/triple lumen catheter and arterial line. 4. Fluid infusion between 80 and 100 mL/kg/d: under whole-body management, fluid control must not be reduced to more than necessary to maintain blood pressure and cerebral circulation. Blood pressure is evaluated using an arterial pressure monitor. Maintenance fluids comprise the prepared acetic acid and lactic acid. Vitamins are administered. When theophylline is administered, vitamin B6 is measured (light-shielding blood collection tube: administer vitamin B6 for theophylline-related seizures. Take care not to induce cardiac arrest by sudden administration of B6). 5. Manage blood count, electrolytes, blood sugar, albumin, and clotting value. Submit to check ferritin, soluble IL-2R (sIL-2R), β2MG, procalcitonin, immunoglobulin, etc. 6. Mannitol 3–5 mL/kg × 4–6 times/d is administered over 1 h. 7. Harvest spinal fluid (after the first administration of mannitol). Submit to check the general spinal fluid, various cytokines (IL-6, IL-1β, and TNF-α), and tau protein. Freeze and store the remaining fluid at −80 °C. 8. If possible, implement MRI (DWI/ADC-map) time-wise. 9. Intratracheal intubation (if difficult, use muscle relaxant or inhalation anesthetic). 10. Artificial ventilation: use pCO_2_ at 35–40 mmHg (do not over-ventilate). Keep PEEP slightly low considering brain hypertension. Raise head by 10°. If brain hypertension occurs, request placement of intracerebral pressure (ICP) monitor by a neurosurgeon. 11. Steroid pulse therapy: use methylprednisolone 30 mg/kg for >2 h for 3 d, during which heparin or fragmin therapy is continued ≥APTT 1.5. 12. Administer famotidine 0.5 mg/kg twice/day or omeprazole (15 years and older 20 mg twice/day). 13. Brain hypothermia therapy: use the whole-body blanket-cooling method to induce target body temperature (direct intestine or bladder temperature of 34.0 °C–35.0 °C or 35.5 °C) within 6 h of onset. If necessary, cool the head or wash the stomach with normal saline while taking care not to cause electrolyte abnormalities or use chilled fluid infusion. Brain hypothermia is managed in the ICU followed by TTM in the ICU or intensively manageable general ward. TTM of maintaining brain temperature between 36 °C and 37 °C using bladder temperature as an indicator, with a high correlation. 14. Antiseizure medication: use sodium thiopental, 5–10 mg/kg/h (if this cannot be used, consider midazolam, 0.3–0.9 mg/kg/h). 15. Mitochondrial rescue therapy (MRT): use coenzyme Q10 3–5 mg/kg/day, Vitamin B2 5 mg/kg/day, Vitamin C 50 mg/kg/day, Vitamin H 0.5 mg/kg/day, Vitamin B1 10 mg/kg/day, Vitamin B6 20–50 mg/kg/day, Vitamin B12 0.03–0.05 mg/kg/day, Vitamin E 10 mg/kg/day, and Carnitine 50–100 mg/kg/day. The duration of treatment should be determined by the clinical course. It is usually 2 weeks or more. 16. Dexstromethorphan therapy: use 2 mg/kg for 5 days for prevention of biphasic encephalopathy after febrile status convulsive. Start within 6 h of onset of convulsion. 17. Sedation depth should be confirmed by portable electroencephalograph or paperless electroencephalograph (Makin2) as reaching suppression burst within 6 h of beginning therapy. Cooling period 18. Maintain the target temperature for 48 h (or a maximum of 72 h) in the ICU or general ward. Confirm BIS value at suppression burst (aim for 40 or below) and adjust the sodium thiopental dose administered based on BIS value as appropriate. Cases achieving positive sedation depth should have their sodium thiopental dose reduced prior to rewarming at BIS values between 60 and 70, and at a body temperature of 35.0 °C. [Caution] If spikes remain with suppression bursts, consider complete suppression (pupils will constrict to mydriasis, and response to light is lost with a BIS value of 20 or lower). 19. Use INVOSTM at an appropriate time to check oxygen saturation at the left and right front scalp and to evaluate brain circulation. 20. Blood pressure maintenance: use an appropriate dose of dopamine hydrochloride (5 µg/kg/min = 0.3 mg/kg/h = 0.015 mL/kg/h) and manage electrolyte abnormalities and blood glucose. Heart rate will fall to bradycardia with falling body temperature. 21. Administer antibacterial as appropriate. In applicable conditions, cerebroprotective edaravone, sivelestat Na as a neutrophil elastase inhibitor, and acyclovir are administered. Rewarming period 22. Rewarming is performed at a rate of 0.5 °C/12 h. Care should be taken to avoid pneumonia in line with increased sputum secretions. Aim to remove the patient from artificial respiration between days 5 and 7. For cases in which laryngitis is likely, intravenous dexamethasone or epinephrine should be administered prior to the removal of the tube. 23. For cases in which critical complications are envisaged, TRH therapy should be initiated at an early stage. 24 Include rehabilitation, aiming to discharge the patient one month after onset. 25. Prior to discharge, evaluate brain waves, implement neuroradiological images or nuclear medicine tests, and assess development. Where necessary, antiseizure mediation should be periodically administered for preventative purposes.

## Data Availability

Not applicable.
